# Rhinovirus-Induced Cytokine Alterations With Potential Implications in Asthma Exacerbations: A Systematic Review and Meta-Analysis

**DOI:** 10.3389/fimmu.2022.782936

**Published:** 2022-02-15

**Authors:** Kong Yen Liew, Sue Kie Koh, Suet Li Hooi, Matthew Kah Lup Ng, Hui-Yee Chee, Hanis Hazeera Harith, Daud Ahmad Israf, Chau Ling Tham

**Affiliations:** ^1^ Department of Biomedical Sciences, Faculty of Medicine and Health Sciences, Universiti Putra Malaysia, Serdang, Malaysia; ^2^ School of Science, Monash University Malaysia, Subang Jaya, Malaysia; ^3^ Department of Medical Microbiology, Faculty of Medicine and Health Sciences, Universiti Putra Malaysia, Serdang, Malaysia

**Keywords:** rhinovirus, asthma, exacerbation, cytokine, chemokine, interferon, systematic review, meta-analysis

## Abstract

**Background:**

Rhinovirus (RV) infections are a major cause of asthma exacerbations. Unlike other respiratory viruses, RV causes minimal cytotoxic effects on airway epithelial cells and cytokines play a critical role in its pathogenesis. However, previous findings on RV-induced cytokine responses were largely inconsistent. Thus, this study sought to identify the cytokine/chemokine profiles induced by RV infection and their correlations with airway inflammatory responses and/or respiratory symptoms using systematic review, and to determine whether a quantitative difference exists in cytokine levels between asthmatic and healthy individuals *via* meta-analysis.

**Methods:**

Relevant articles were obtained from PubMed, Scopus, and ScienceDirect databases. Studies that compared RV-induced cytokine responses between asthmatic and healthy individuals were included in the systematic review, and their findings were categorized based on the study designs, which were *ex vivo* primary bronchial epithelial cells (PBECs), *ex vivo* peripheral blood mononuclear cells (PBMCs), and human experimental studies. Data on cytokine levels were also extracted and analyzed using Review Manager 5.4.

**Results:**

Thirty-four articles were included in the systematic review, with 18 of these further subjected to meta-analysis. Several studies reported the correlations between the levels of cytokines, such as IL-8, IL-4, IL-5, and IL-13, and respiratory symptoms. Evidence suggests that IL-25 and IL-33 may be the cytokines that promote type 2 inflammation in asthmatics after RV infection. Besides that, a meta-analysis revealed that PBECs from children with atopic asthma produced significantly lower levels of IFN-β [Effect size (ES): -0.84, *p* = 0.030] and IFN-λ (ES: -1.00, *p* = 0.002), and PBECs from adult atopic asthmatics produced significantly lower levels of IFN-β (ES: -0.68, *p* = 0.009), compared to healthy subjects after RV infection. A trend towards a deficient production of IFN-γ (ES: -0.56, *p* = 0.060) in PBMCs from adult atopic asthmatics was observed. In lower airways, asthmatics also had significantly lower baseline IL-15 (ES: -0.69, *p* = 0.020) levels.

**Conclusion:**

Overall, RV-induced asthma exacerbations are potentially caused by an imbalance between Th1 and Th2 cytokines, which may be contributed by defective innate immune responses at cellular levels. Exogenous IFNs delivery may be beneficial as a prophylactic approach for RV-induced asthma exacerbations.

**Systematic Review Registration:**

https://www.crd.york.ac.uk/prospero/display_record.php?RecordID=184119, identifier CRD42020184119.

## Introduction

Rhinovirus (RV) is a positive sense, single-stranded RNA virus (+ssRNA) that belongs to the Picornaviridae family. RV can be classified into three different species based on sequence homology: RV-A, RV-B, and RV-C, with more than 160 serotypes already identified (1). RV infections account for more than half of the common colds. They are a major cause of exacerbations of chronic respiratory diseases such as asthma, chronic obstructive pulmonary disease (COPD), and cystic fibrosis (CF). Currently, there are no approved drugs to prevent or treat RV infections (2). Thus, an increased understanding of the pathogenesis of RV infection is needed to develop effective therapeutic approaches.

In epidemiological studies, RV infections were frequently found to be associated with a majority of exacerbations of asthma in adults as well as children. A recent meta-analysis by Feddema and Claassen (3) has shown that RV is the most commonly detected virus in asthmatics, contributing up to 45.6% and 68.5% of virus-induced acute asthma exacerbations cases in adults and children, respectively. Besides that, Corne and colleagues (4) have demonstrated that while asthmatic and healthy subjects in a longitudinal cohort study had a similar frequency of upper respiratory RV infections, asthmatic subjects had more frequent, severe, and longer-lasting lower respiratory tract symptoms. Several experimental RV infection studies supported the causal relationship between RV infection and asthma exacerbations. Experimental RV infection of asthmatics has been reported to increase bronchial hyperresponsiveness, airway obstruction, and airway inflammation compared to the placebo group without RV inoculation (5–7). Furthermore, asthmatic individuals experienced more significant lower respiratory tract symptoms, a decline in lung function, increased bronchial hyperresponsiveness, and eosinophilic lower airway inflammation compared with healthy individuals. These symptoms appear to be related to the viral load and an imbalance between T helper type 1 (Th1) and Th2 cytokine levels (8). Collectively, these findings indicate that RV is a significant respiratory pathogen in asthmatic individuals.

Although the pathogenesis of RV-induced asthma exacerbations is not yet fully elucidated, it is linked to the host immune responses triggered by RV infections. RV primarily targets the airway epithelial cells in the upper and lower respiratory tracts (9). However, unlike other respiratory viruses, RV does not cause overt cytotoxicity *in vitro* or *in vivo* (10). It binds to intercellular adhesion molecule-1 (ICAM-1) (for major group RV), low-density lipoprotein receptor (LDLR) family members (for minor group RV) or cadherin-related family member 3 (CDHR3) (for RV-C) to gain entry to the target cells (11). The detection of the virus by the epithelial pattern recognition receptors (PRRs) such as toll-like receptor 3 (TLR3), melanoma differentiation-associated gene 5 (MDA5), and retinoic acid-inducible gene I (RIG-I) triggers the production of a variety of cytokines by the infected epithelial cells (12). These include proinflammatory cytokines/chemokines, such as interleukin (IL)-6, IL-8, interferon gamma-induced protein 10 (IP-10), regulated upon activation, normal T Cell expressed and secreted (RANTES), granulocyte-macrophage colony-stimulating factor (GM-CSF), monocyte chemoattractant protein-1 (MCP-1), interferon (IFN)-β, and IFN-λ. The proinflammatory cytokines and chemokines subsequently recruit different inflammatory cells, such as neutrophils, monocytes, and lymphocytes, which also release cytokines, chemokines, matrix metalloproteinases (MMPs), and reactive oxygen species (ROS) upon activation, resulting in an enhanced inflammatory response within the respiratory tract (10).

Other than modulating epithelial cell responses, RV has been shown to alter the immune responses of monocytic cells such as monocytes, macrophages, T lymphocytes, and mast cells (13–16). The release of cytokines, such as IL-1, IL-8, TNF-α, IFN-α, and IFN-γ triggered by RV infection, activates the surrounding cells and induces the expression of adhesion molecules on epithelial cells as well as immune cells. Given the close proximity of monocytic cells to the airway epithelium in the respiratory tract, they could also influence the immune responses to RV infection *in vivo* and contribute to asthma exacerbations (17).

Existing literature suggests that the host immune responses, particularly the cytokine responses induced by RV infections, are linked to the airway inflammatory responses and potentially the severity of asthma exacerbations. Previous studies have also shown that RV infections induce the production of a variety of proinflammatory cytokines/chemokines including IFNs in primary bronchial epithelial cells (PBECs) and peripheral blood mononuclear cells (PBMCs) from asthmatic and healthy individuals. However, these findings were largely inconsistent. Thus, this study aimed to compare the cytokine/chemokine profiles induced by RV infection in asthmatic and healthy individuals and their correlations with viral load, airway inflammatory responses, and/or respiratory symptoms *via* systematic review. Meta-analysis was also performed to determine whether any quantitative difference exists in the levels of cytokines between the asthmatic and healthy groups.

## Methods

### Search Strategy

Relevant articles were identified from three different databases (PubMed, Scopus, and ScienceDirect) using keywords: rhinovirus AND asthma AND (cytokine OR chemokine OR interferon OR antiviral). All studies that compared rhinovirus-induced cytokine responses between asthmatic and healthy individuals were included in this systematic review. There were three types of study designs for related studies, which were (1) *ex vivo* PBECs studies, (2) *ex vivo* PBMCs studies, and (3) human experimental studies. There was no restriction on the publication period, but only articles in English were included in this systematic review. Filter was applied to include original research articles only whenever applicable in the databases. This study was conducted according to the Preferred Reporting Items for Systematic Reviews and Meta-analysis (PRISMA) guidelines (18). The methods used here have also been described in detail in our published protocol in the International Prospective Register of Systematic Reviews (PROSPERO) database (Registration: CRD42020184119). The last search for relevant articles in all databases was performed on 13 August 2021.

### Eligibility Criteria

For this systematic review, the inclusion criteria are (1) *ex vivo* PBECs studies, (2) *ex vivo* PBMCs studies, and (3) human experimental studies that compared RV-induced cytokine responses between asthmatic and healthy individuals. Asthmatic and healthy individuals of all ages and sexes were included. For *ex vivo* or human experimental infection, rhinoviruses of all species or serotypes in any dose were included. On the other hand, the exclusion criteria are (1) animal studies, (2) studies on other respiratory viruses (e.g. respiratory syncytial virus, influenza, parainfluenza, etc.), and (3) studies that did not include a healthy control (without asthma) group.

### Study Appraisal and Selection

All the articles obtained from the databases using the specific keywords were organized according to their titles, and duplicates were identified by the same title, authors, and year of publication. The redundant studies were removed, and the remaining articles were screened using the pre-defined eligibility criteria. Firstly, the title and abstract of each article were assessed by two reviewers independently. Those that matched the eligibility criteria were then subjected to full-text screening to determine their relevance further. Disagreements between the two reviewers throughout the screening process were resolved by consensus, and the reasons for excluding the articles were recorded.

### Data Extraction and Analysis

For all studies that met the eligibility criteria after reading the full texts, study characteristics, such as subjects’ demographics (age and sex) and medication (corticosteroids), smoking, and allergy status, sample size (asthmatic vs healthy), rhinovirus used for infection (species, subtype, and dose), type of sample (culture supernatant or biological fluid), and method of cytokine measurement, were extracted and organized into tables according to their study designs. This was followed by the extraction of findings related to RV-induced cytokine responses and their correlations with viral load, airway inflammatory responses, and/or respiratory symptoms.

To determine whether any quantitative difference exists in the levels of cytokines between the asthmatic and healthy groups, a meta-analysis was carried out to analyse the levels of cytokines between the two groups. Given that three different experimental designs were included in this systematic review, there must be at least two different studies that measured the same cytokine under the same experimental design for the cytokine to be subjected to meta-analysis. The mean levels of these cytokines (pg/mL) and their standard error of mean (SEM) or standard deviation (SD) were extracted from the texts or the graphs using the software ImageJ version 150. For the studies that reported the levels of cytokines using the median, range, and/or interquartile range, estimation of the mean and standard deviation was performed using a previously described method (19). The data were entered into the Excel Spreadsheet with formulas provided that estimates the mean and standard deviation according to three different scenarios for the reporting values: (1) minimum, median, maximum, and sample size; (2) minimum, the first quartile, median, the third quartile, maximum, and sample size; or (3) the first quartile, median, the third quartile, and sample size (19).

Meta-analysis was performed using the software Review Manager (RevMan) version 5.4. Input data for each included study are required to be in the form of mean and SD. If SEM was extracted instead of SD, the values of SEM were converted into SD using the built-in function of the software. The standardized mean difference (SMD) and 95% confidence intervals (CI) of cytokine levels between the asthmatic and healthy groups were computed to obtain the effect size (ES) for each cytokine. The overall effect (*p-value*) was determined using a fixed-effect or random-effects model based on the I-squared index (I^2^) that indicates the heterogeneity between studies. The fixed-effect model was used if the heterogeneity between studies was not significant (I^2^ less than 75%). By contrast, the random-effects model was used if the heterogeneity between studies was significant (I^2^ more than 75%) (20).

### Publication Bias Assessment

Publication bias assessment was performed using Meta-Essentials (21). Cytokine levels (mean and SD) and sample sizes for each study were inserted into Meta-Essentials, and publication bias was evaluated based on the Funnel plot and the results of Egger’s test, Begg’s test, Rosenthal’s Failsafe N, and Trim and Fill method.

## Results

### Study Selection

**Figure 1:**
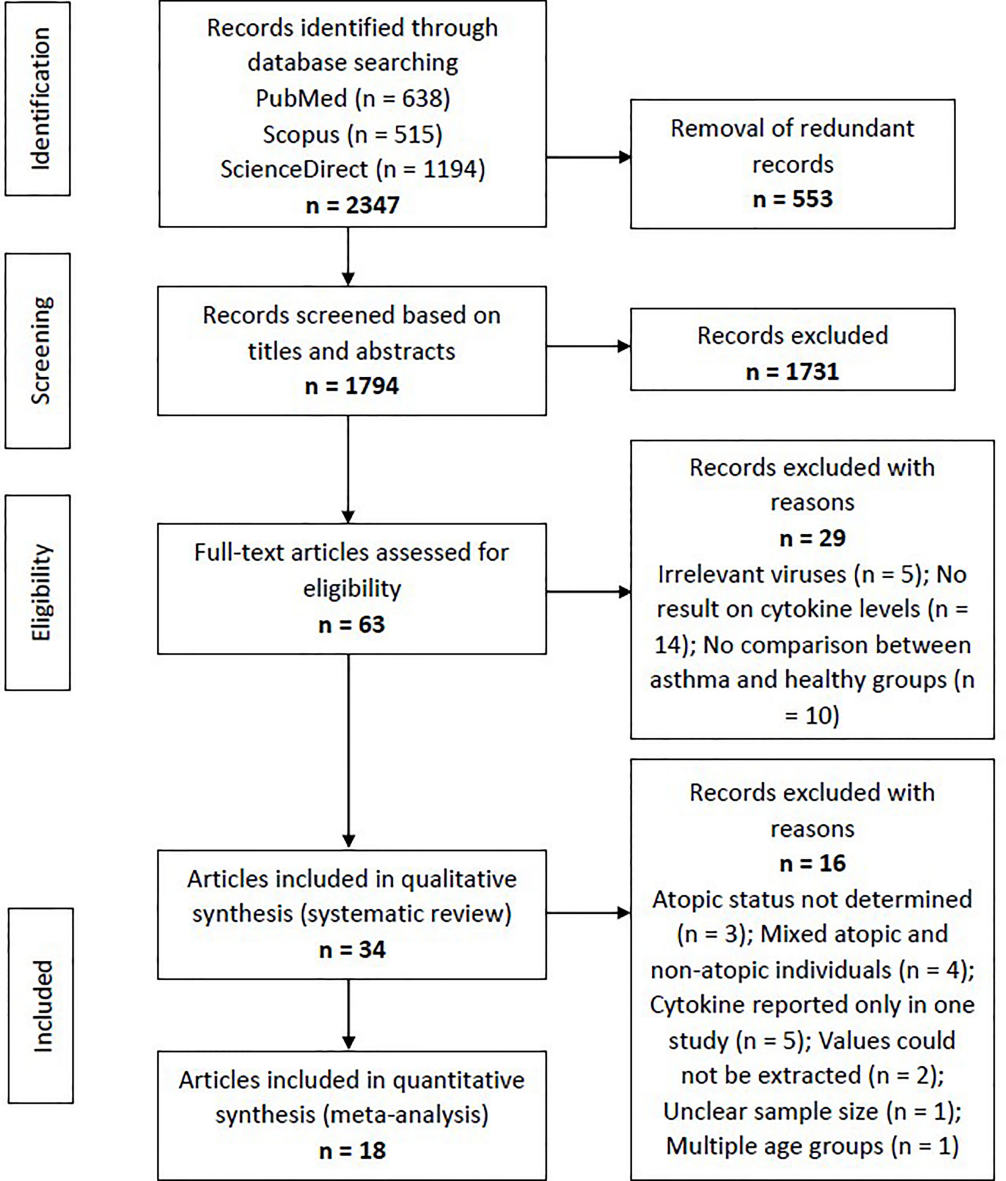
Flowchart showing the selection process of articles according to the PRISMA Statement. From the initial 1794 non-redundant articles, 34 articles reporting RV-induced cytokine responses in asthmatic and healthy individuals were included in the systematic review, with 18 of these further subjected to meta-analysis.

Using the specific keywords, we had retrieved 638 articles from PubMed, 515 articles from Scopus, and 1194 articles from ScienceDirect (n = 2347). After removing the redundant articles (n = 553), the number of unique articles screened based on the titles and abstracts was 1794. Sixty-three articles were subjected to full-text screening to assess their suitability based on the eligibility criteria. Twenty-nine articles were excluded due to several reasons, such as irrelevant viruses (n = 5), no result on cytokine levels (n = 14), and no comparison between asthma and healthy groups (n = 10). Finally, 34 articles reporting RV-induced cytokine responses in asthmatic and healthy individuals were included in the systematic review. **Figure 1** shows the PRISMA flowchart for study selection.

The included articles were separated according to their experimental designs into three different categories, which were (1) *ex vivo* PBECs studies, (2) *ex vivo* PBMCs studies, and (3) human experimental studies. Some articles involved more than one experimental design (e.g. *ex vivo* PBECs and human experimental studies in one article). Taking this into account, there are 18 *ex vivo* PBECs studies, 10 *ex vivo* PBMCs studies, and 10 human experimental studies reported by the 34 articles. Of these 34 articles, 18 articles were included in the meta-analysis (**Figure 1**).

### Study Characteristics

All studies were conducted in the adult populations, except 3 PBECs studies and 1 PBMCs study, which compared the cytokine responses between asthmatic and healthy children after RV infection (22–25), as well as 1 PBMCs study which compared RV-induced cytokine responses between asthmatic and healthy individuals from different age groups (26). For most of the included studies, the asthmatic subjects were atopic with positive skin prick test (SPT) or had increased levels of specific IgE to allergens, whereas the healthy subjects were non-atopic. A study by Baraldo et al. (22) made comparisons between 4 groups of subjects (atopic asthma vs non-atopic asthma vs atopic healthy vs non-atopic healthy), and a study by Moskwa et al. (27) also made an additional comparison between atopic asthma and non-atopic asthma. For the studies by Iikura et al. (26), Jurak et al. (28), and Williams et al. (29), the authors compared RV-induced cytokine responses between asthmatic and healthy individuals; however, both asthma and healthy groups included atopic and non-atopic individuals. Apart from that, the asthmatic subjects in a study by Hosseini et al. (25) consisted of both atopic and non-atopic individuals. The atopic status of both asthmatic and healthy subjects was not determined in 3 studies (30–32). Notably, the atopic status may be a potential confounding factor of RV-induced cytokine responses (further discussed in the section below). Asthmatic subjects mainly had mild or moderate asthma, but some studies recruited severe asthma patients (23, 27, 32–35). Most of the studies also excluded subjects who had smoking habits, probably due to previously reported effects of cigarette smoke on RV-induced cytokine production or innate immune responses (36–38). The subject characteristics of all included studies are provided in **Supplementary Tables 1–3**, whereas the experimental details are provided in **Supplementary Tables 4–6**.

### Systematic Review

#### Cytokine/Chemokine Profiles Induced by RV Infection in Asthmatic and Healthy PBECs

At least two studies have reported significantly increased levels of proinflammatory cytokines and chemokines in both asthmatic and healthy PBECs following RV infection. These cytokines/chemokines include IL-1α (32, 39), IL-6 (24, 32, 34, 39–42), IL-8 (24, 32, 34, 41, 42), TNF-α (32, 41), IP-10 (24, 27, 32, 34, 39, 41), and RANTES (24, 27, 32, 39–41), and interferons, such as IFN-β (22, 40, 43, 44) and IFN-λ (27, 33, 34, 44) (**Table 1**). However, inconsistent results have been described for some of these cytokines. While most studies observed similar induction of IL-6 (32, 39–42), IL-8 (32, 41, 42), IP-10 (27, 32, 34, 39, 41), and RANTES (27, 32, 39–41) in both asthma and healthy groups, some studies have reported that these cytokines were only induced in asthmatic PBECs (23, 30). Cakebread et al. (30) reported that RV-1B only significantly induced IL-6, IP-10, and RANTES in asthmatic but not healthy PBECs, while Edwards et al. (23) reported that RV-1B only significantly induced IL-8 in asthmatic PBECs. By contrast, a few studies demonstrated that asthmatic PBECs produced significantly lower levels of cytokines such as IL-6 (34), IL-8 (34), TNF-α (35), and IP-10 (35) compared to healthy PBECs.

**Table 1:** Findings of *ex vivo* PBECs studies comparing RV-induced cytokine responses in asthmatic vs healthy individuals.

RV-induced cytokine responses	Cytokine/Chemokine	References
Significant up-regulation in both asthmatic and healthy PBECs compared to mock-infected control	IL-1α	(32, 39)
	IL-6	(24, 32, 34, 39, 40, 42, 45)
	IL-8	(24, 32, 34, 42, 45)
	IP-10^1^	(24, 27, 32, 34, 39, 45)
	RANTES^1^	(24, 27, 32, 39, 40, 45)
	TNF-α	(32, 45)
	IFN-β^2^	(22, 40, 43, 44)
	IFN-λ	(27, 33, 34, 44)
Significantly higher levels in asthmatic PBECs compared to healthy PBECs	IL-1β	(24)
	IL-6	(24)
	IL-8	(24)
	IP-10	(24)
	RANTES	(24)
	IL-25	(46)
	TGF-β2	(31)
Significantly lower levels in asthmatic PBECs compared to healthy PBECs	IL-6	(34)
	IL-8	(34)
	IP-10^1^	(35)
	TNF-α	(35)
	IFN-β^1^	(23, 24, 40, 43)
	IFN-λ^1,2,3^	(23, 24, 33, 34)

^1^Cytokine/chemokine correlated with vRNA or infectious virus levels.

^2^Cytokine/chemokine correlated with airway inflammatory responses (e.g. eosinophil counts, proinflammatory cytokine levels in bronchial biopsies or airway fluids).

^3^Cytokine/chemokine correlated with respiratory symptoms (e.g. cold score, airway hyperresponsiveness, reduced lung function).

Besides that, contradicting data on IFNs (IFN-β and IFN-λ) production in asthmatic and healthy PBECs have been reported. Impaired IFNs production in asthmatic PBECs has been reported by six studies (23, 24, 33, 34, 40, 43), in which RV infection caused a significantly lower induction of IFN-β (23, 24, 40, 43) and IFN-λ (23, 24, 33, 34) protein levels in asthmatic PBECs in comparison to healthy PBECs; however, such changes were not observed by three other studies (22, 27, 44). Nonetheless, it should be noted that some studies were not able to detect IFN-β and IFN-λ in the PBEC cultures of both asthmatic and healthy individuals (22, 27, 35). Apart from that, PBECs do not seem to produce a significant amount of IFN-α protein as three (24, 27, 44) out of four studies (24, 27, 32, 44) that measured IFN-α did not detect it in the culture supernatants of asthmatic and healthy PBECs.

Notably, some studies reported a differential response to RV infection between asthmatic and healthy PBECs for some relatively less studied cytokines such as IL-25 and TGF-β2. Asthmatic PBECs were found to produce significantly higher levels of IL-25 (46) and TGF-β2 (31) after RV infection compared to healthy PBECs. Collectively, these findings indicate that PBECs isolated from asthmatic individuals do not share a similar RV-induced cytokine/chemokine profile with PBECs isolated from healthy individuals.

#### Cytokine/Chemokine Profiles Induced by RV Infection in Asthmatic and Healthy PBMCs

Overall, at least two studies have demonstrated that RV infection significantly induces IL-6 (26, 47), IL-10 (26, 47, 48), IP-10 (49, 50), IFN-γ (47, 48, 51), and IFN-α (26, 44, 47, 49, 50) in both asthmatic and healthy PBMCs (**Table 2**). Similar to the PBEC studies, deficient IFNs production in PBMCs from asthmatic individuals has been reported. However, these reports focused on different types of IFN, namely IFN-γ and IFN-α. Impaired IFN-γ production in asthmatic PBMCs compared to healthy PBMCs has been demonstrated by a few groups of researchers in adult populations (28, 48, 51) and children (25). By contrast, some studies reported similar levels of IFN-γ between asthma and healthy groups after RV infection (26, 47). Inconsistent findings were also observed for IFN-α. RV-induced IFN-α levels in PBMCs were reported to be significantly lower in the asthma group than in the healthy group (26, 50). In particular, Iikura et al. (26) found that the significant difference was restricted to asthmatics between 7 to 19 years old and their age-matched healthy controls but not for 20 years old and above, indicating that the age of donors may also be a determinant of RV-induced cytokine responses. Conversely, other studies did not significantly differ between asthma and healthy groups on IFN-α production (49, 52). Besides IFNs, IL-10 is another cytokine that may be differentially expressed in asthmatic and healthy PBMCs in response to RV infection. Papadopoulos et al. (48) showed that RV-16 infection led to a significantly higher level of IL-10 in PBMCs of asthmatic subjects compared to healthy subjects. This finding is in line with another study by Xatzipsalti et al. (51), which showed that IL-10 protein was only significantly induced in asthmatic PBMCs. However, other studies have also reported lower amounts of IL-10 (26) in asthmatic PBMCs than healthy PBMCs or no difference (47). These findings indicate that differential cellular responses in terms of cytokine production between asthmatic and healthy individuals following RV infection may not only be limited to PBECs.

**Table 2:** Findings of *ex vivo* PBMCs studies comparing RV-induced cytokine responses in asthmatic vs healthy individuals.

RV-induced cytokine responses	Cytokine/Chemokine	References
Significant up-regulation in both asthmatic and healthy PBMCs compared to mock-infected control	IL-6	(26, 47)
	IL-10	(26, 47, 48)
	IP-10	(49, 50)
	IFN-α^1^	(26, 47, 49, 50, 52)
	IFN-γ	(26, 48, 51)
Significantly higher levels in asthmatic PBMCs compared to healthy PBMCs	IL-1β	(25)
	IL-10	(48)
	Fractalkine	(53)
Significantly lower levels in asthmatic PBMCs compared to healthy PBMCs	IL-6	(26)
	IL-10	(26)
	IL-12	(48)
	TNF-α	(26)
	IFN-α	(26, 50)
	IFN-γ	(25, 28, 48, 51)

^1^Cytokine/chemokine correlated with respiratory symptoms (e.g. cold score, airway hyperresponsiveness, reduced lung function). For PBMCs studies, no cytokine/chemokine was reported to be correlated with vRNA/infectious virus levels and airway inflammatory responses.

#### Levels of IFNs Produced by PBECs and PBMCs Correlate With Symptoms and Disease Severity of RV Infection and/or RV-Induced Asthma Exacerbations

Among the cytokines and chemokines induced by RV, as described in previous sections, some have been shown to play an essential role in the pathogenesis of RV infection. A study by Contoli et al. (33) demonstrated that PBECs, as well as bronchoalveolar lavage (BAL) cells (mainly macrophages), had a deficient production of IFN-λ after RV-16 infection *ex vivo*, and this was related to disease severity in the donors who were experimentally infected with RV after the *ex vivo* study. IFN-λ protein levels in BAL cells were inversely correlated with the cold score, viral load, IL-8 levels in BAL fluid, sputum eosinophil counts, and a fall in the forced expiratory volume in one second (FEV1) (33). These findings suggest an association between low IFN-λ level and increased viral replication and inflammatory responses in the respiratory tract, cold symptoms, and reduced lung function. Other than IFN-λ, deficient IFN-β production in PBECs is also linked to RV-induced pathological changes in airways. A study by Baraldo et al. (22) demonstrated that RV-16-induced IFN-β levels are inversely correlated with the expression of IL-4 in bronchial biopsies and the degree of epithelial damage, which are common characteristics of asthmatic airways. Furthermore, viral RNA (vRNA) and infectious virus levels in culture supernatants of RV-infected PBECs were negatively correlated with the levels of IFN-β and IFN-λ, suggesting a protective role for both cytokines against RV infection by suppressing the replication rate of RV (22, 33, 35, 43). This is supported by another study which demonstrated that low levels of IFN-α and IFN-β in PBMCs are associated with more significant airway hyperresponsiveness, as indicated by lower provocative concentration of histamine causing a 20% fall in FEV1 (PC20) (52).

#### Influence of Atopic Status on RV-Induced Cytokine Responses in PBECs

While most of the studies suggest that the presence of asthma may be the factor contributing to the differential cytokine responses of PBECs and PBMCs to RV infection, a few studies suggested that the atopic status of the subjects could influence RV-induced cytokine responses. Baraldo et al. (22) found that PBECs from atopic asthma, non-atopic asthma, and healthy atopic children had impaired IFN-β and IFN-λ induction in response to RV infection compared to PBECs from non-atopic healthy children. As PBECs from healthy atopic children also showed defective production of IFNs, which was similarly observed in asthmatic PBECs, this suggests that other than the presence of asthma, the atopic status of the PBECs donors could affect their cytokine responses to RV infection. In line with this finding, some studies found correlations between cytokine levels and the number of positive skin prick tests (SPT). For instance, IL-25 levels in PBECs were positively correlated with positive SPT in asthmatics (46). By contrast, IFN-α and IFN-β levels in PBMCs were negatively correlated with positive SPT in asthmatics (52). In comparison to PBECs, all PBMCs and human experimental studies included in this study (**Tables 2**, **3**) compared RV-induced cytokine responses between atopic asthmatics and non-atopic healthy individuals only, except for two *ex vivo* PBMCs studies that recruited both atopic and non-atopic subjects in asthma and healthy groups (26, 27). Thus, no direct comparisons on the cytokine levels were made between atopic and non-atopic individuals among asthma and healthy groups (atopic asthma vs non-atopic asthma vs atopic healthy vs non-atopic healthy) for these two study designs (*ex vivo* PBMCs and human experimental studies). In order to avoid potential confounding effects of atopic status on RV-induced cytokine responses, allergy tests such as SPT and tests on allergen-specific IgE should be conducted to allow stratification of the subjects according to their atopic status. Furthermore, as *ex vivo* PBECs studies indicate that atopic status could influence RV-induced cytokine responses, it would also be interesting to investigate whether natural or experimental RV infection will lead to more severe respiratory symptoms in atopic compared to non-atopic individuals for asthmatic as well as healthy subjects in the future.

**Table 3:** Findings of human experimental studies comparing nasal and bronchial cytokine responses in asthmatic vs healthy individuals.

Upper and lower airway cytokine responses	Subjects	Significantly upregulated cytokine/chemokine
Upper airway (nasal lavage, nasal mucosal fluid)	Asthma	IL-2^*^ (54), IL-4*^,#^ (55), IL-5^3,^*^,#^ (54, 55), **IL-6^2^** (47, 54, 56), **IL-8 ^1,2,3^** (47, 57), IL-10 (47, 54), IL-12p40 (54), IL-13^3,^*^,#^ (54, 55), IL-15^1,*^ (54), IL-17 (54), IL-25 (46), **IL-33^1,3^** (54, 55), IP-10^*^ (54), RANTES (47, 54), TNF-α (54), MCP-1^1,*^ (47, 54), MIP-1α^1^ (54), MIP-1β^1^ (54), MIP-3α (54), ITAC^*^ (54), TARC^2,3,^*^,#^ (29, 54), MDC*^,#^ (29, 54), eotaxin*^,#^ (54), IFN-α (47), IFN-γ^*^ (47, 54), IFN-λ^*^ (54)
	Healthy	IL-6 (47, 56), IL-8 (47, 56), IL-25 (46), RANTES (47), MCP-1 (47), IFN-α (47), IFN-γ (47)
Lower airway (bronchial mucosal fluid, BAL fluid, sputum)	Asthma	**IL-5^3^** (54, 55), IL-6 (56), **IL-8^1,#^** (58), IL-10 (47, 54), IL-15 (54), IP-10 (54), TNF-α (54), ITAC (54), IFN-γ (54)
	Healthy	IL-6 (56), IL-8 (56), **IL-15^1,3,#^** (59)

^1^Cytokine/chemokine correlated with nasal or bronchial viral load.

^2^Cytokine/chemokine correlated with airway inflammatory responses (e.g. eosinophil or neutrophil counts, proinflammatory cytokine levels in airway fluids).

^3^Cytokine/chemokine correlated with respiratory symptoms (e.g. upper or lower respiratory symptom score, airway hyperresponsiveness, reduced lung function).

^*^Cytokine differentially expressed by asthmatic subjects following experimental RV infection compared to healthy subjects.

^#^Cytokine with significant baseline differences.

#### Nasal and Bronchial Cytokine Responses Following Experimental RV Infection and Their Associations With Pathophysiological Mechanisms of RV Infection and/or RV-Induced Asthma Exacerbations

Inoculation of human subjects with RV has been shown to cause inflammatory changes in both the upper (nasal) and lower (bronchial) airways as indicated by increased levels of proinflammatory cytokines in the nasal lavage, nasal mucosal fluid, sputum, BAL fluid, or bronchial mucosal fluid (**Table 3**). IL-6 and IL-8 levels were increased in the nasal lavage/fluid, sputum, and BAL fluid of both asthmatic and healthy subjects post-RV inoculation (47, 56, 58, 60). Moreover, positive correlations between nasal IL-8 levels and neutrophil counts (47, 56), peak viral load (58), as well as cold symptoms (47, 56) have been previously reported. These findings are consistent with its role as a chemokine that attracts neutrophils.

Allergic asthma is characterized as type 2 inflammation associated with Th2 cytokines such as IL-4, IL-5, and IL-13 (61). These cytokines also seem to play a crucial role in the pathogenesis of RV-induced asthma exacerbations. A human experimental study by Hansel et al. (54) demonstrated that experimental RV infection in people with allergic asthma is accompanied by more intense type 2 inflammatory responses (IL-5 and IL-13) compared to healthy subjects with varying degrees of antiviral responses (IFN-γ and ITAC). The same study reported that experimental RV infection caused significantly greater upper and lower respiratory symptoms and reductions in peak expiratory flow (PEF) and FEV1 in asthmatic subjects compared to healthy subjects (55). A causative role for IL-4, IL-5, and IL-13 in RV-induced respiratory symptoms was proposed as these cytokines were significantly upregulated in the nasal mucosal fluid of asthmatics but not healthy subjects (55). Most importantly, nasal and bronchial IL-5 and IL-13 levels were also shown to be positively correlated with the severity of upper and lower respiratory symptoms in asthmatic subjects (55). The same study also presented strong evidence that highlights a role for IL-33 in promoting type 2 inflammatory responses in RV-induced asthma exacerbations. Not only did bronchial IL-33 levels correlate with the levels of IL-5 and IL-13, nasal and bronchial IL-33 levels also positively correlate with asthma symptom severity, suggesting that IL-33 promotes Th2 cytokine responses that subsequently drive airway inflammation in asthma. Accordingly, exposure of Th0 cells to supernatants from RV-infected PBECs, which contained a significantly upregulated level of IL-33, induced Th2 responses with increased IL-4, IL-5, and IL-13 compared to Th0 cells exposed to supernatants from non-infected PBECs. Blocking the action of IL-33 using anti-ST2 antibody inhibited IL-4, IL-5, and IL-13 secretion in Th0 cells exposed to supernatants from RV-infected PBECs, suggesting that RV-induced Th2 responses are IL-33-dependent (55). In agreement with these findings, a study by Jurak et al. (28) also demonstrated that IL-33 selectively enhanced RV-induced IL-5 and IL-13 release in PBMCs of asthmatic but not healthy individuals. Besides IL-33, IL-25 has also been claimed to be an essential Th2-promoting cytokine. Beale et al. (2014) reported that IL-25 is significantly induced by RV infection in asthmatic PBECs compared to mock-infected control but not in healthy PBECs (46). However, the same study further demonstrated that the difference between asthmatic and healthy individuals in the nasal IL-25 levels was insignificant, albeit much higher in the asthmatics (46). Other than the proinflammatory Th2 cytokines (IL-4, IL-5, and IL-13), a recent study by Williams et al. (29) highlighted the role of chemokines, C-C motif chemokine 17 (CCL17)/thymus- and activation-regulated chemokine (TARC) and C-C motif chemokine 22 (CCL22)/macrophage-derived chemokine (MDC) in RV-induced asthma exacerbations. The study reported that peak nasal TARC and MDC levels positively correlated with peak upper respiratory symptom scores. In addition, bronchial MDC levels on day 4 post-infection positively correlated with peak lower respiratory symptom scores in subjects experimentally infected with RV.

While most studies identified a link between increased levels of cytokines and RV-induced pathophysiological mechanisms in asthma, Laza-Stanca et al. (59) has described the possible role of IL-15 deficiency in RV-induced asthma exacerbations. They reported that IL-15 levels induced by RV infection were significantly lower in BAL cells and BAL fluid of asthmatics compared to healthy subjects (59). Besides that, IL-15 levels in supernatants of BAL cells inversely correlated with the severity of lower respiratory symptoms. By contrast, low baseline IL-15 levels in BAL fluids were associated with more significant airway hyperresponsiveness and higher viral load in nasal lavage, sputum, and BAL fluid (59). Notably, the authors claimed that IL-15 inducer such as IFN-β may have therapeutic benefits as it could promote antiviral immunity of airway epithelium and, at the same time, enhance innate and acquired immunity against RV infection *via* induction of IL-15. This is because IL-15 promotes natural killer (NK) cell activation, increases memory CD8 T cell antiviral immunity, and enhances IFNs production in various cell types such as macrophages, dendritic cells, NK cells, and CD8 T cells (59). Overall, the pathophysiological mechanisms of RV-induced asthma exacerbations involve a complex interplay of airway epithelium, immune cells, and their secreted cytokines in response to RV infection.

#### Viral Load in Asthmatic and Healthy Subjects

Besides cytokine levels, viral load is another factor frequently associated with the severity of RV infection and/or RV-induced asthma exacerbations. Message et al. (8) demonstrated that nasal viral load is significantly correlated with lower respiratory symptoms in asthmatic subjects. Besides that, a strong inverse correlation between viral load and airway hyperresponsiveness in asthma was also observed (8). This is consistent with another study by Jackson et al. (55), which reported that peak viral load is correlated with exacerbation severity (indicated by peak reductions in PEF) in asthmatic subjects. However, there has been considerable debate on whether RV can replicate at a higher rate in the airway epithelium of asthmatics compared to healthy individuals. This observation was first reported by Wark et al. (40), where RV-16 vRNA and infectious virus levels were significantly higher in asthmatic PBECs than healthy PBECs. Similarly, other studies showed that vRNA and infectious virus levels were significantly higher in asthmatic PBECs after RV-16 or RV-1B infection (22–24). By contrast, several studies reported no significant difference between the viral load in PBECs isolated from asthmatic and healthy subjects (30, 32, 35, 39, 42, 44). Similar levels of viral load in nasal lavage fluid and sputum between asthmatic and healthy subjects after experimental RV infection have also been reported (47). However, it may not be appropriate to compare the viral load at the same time point due to the possibility of a different kinetic of RV replication in asthmatic and healthy subjects (55). Jackson and colleagues (55) have demonstrated that nasal viral load peaked one day earlier in asthmatics (day 3 post-inoculation) compared to healthy individuals (day 4 post-inoculation). In contrast to this finding, Kennedy et al. (62) demonstrated that neither peak nor cumulative nasal viral loads in asthmatic adults differed significantly from healthy individuals following experimental RV infection. The differences in viral loads might be due to the different levels of cytokines such as IFN-β and IFN-λ, which have been shown to suppress RV replication. Nevertheless, it is also possible that RV-induced asthma exacerbations are caused by inflammatory responses triggered by RV rather than a higher viral load in asthmatic airways (62).

### Meta-Analysis

Due to the discrepancies across studies, cytokines with at least two studies (for each experimental design) were then subjected to meta-analysis to determine whether asthmatic and healthy subjects produce different levels of cytokines in response to RV infection. However, only studies that compared atopic asthmatic and non-atopic healthy subjects were included to avoid the potential confounding effects of atopic status on cytokine responses.

#### Deficient IFN-β and IFN-λ Production in Atopic Asthmatic PBECs After RV Infection

**Figure 2:**
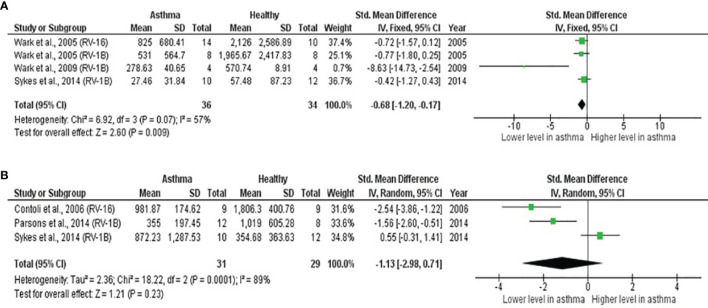
Forest plots of **(A)** IFN-β and **(B)** IFN-λ for *ex vivo* PBECs studies comparing adults with atopic asthma vs non-atopic healthy controls. **(A)** PBECs from adults with atopic asthma produced significantly lower levels of IFN-β (ES: -0.68, *p* = 0.009) than non-atopic healthy subjects after RV infection. **(B)** PBECs from adults with atopic asthma also produced lower levels of IFN-λ (ES: -1.13) compared to non-atopic healthy subjects after RV infection; however, the difference was not statistically significant (*p* = 0.230).

**Figure 3:**
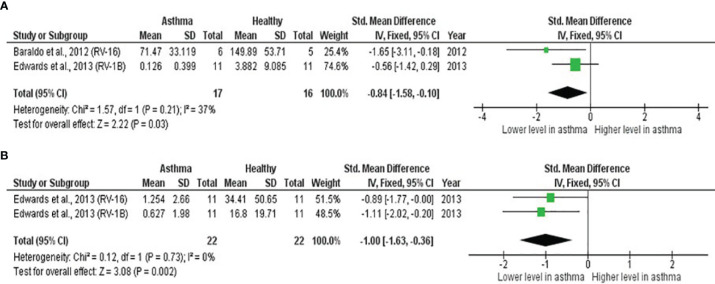
Forest plots of **(A)** IFN-β and **(B)** IFN-λ for *ex vivo* PBECs studies comparing children with atopic asthma vs non-atopic healthy controls. PBECs from children with atopic asthma produced significantly lower levels of **(A)** IFN-β (ES: -0.84, *p* = 0.030) and **(B)** IFN-λ (ES: -1.00, *p* = 0.002) compared to non-atopic healthy children after RV infection.

As shown in **Table 4**, PBECs from adults with atopic asthma produced significantly lower levels of IFN-β (ES: -0.68, *p* = 0.009) than non-atopic healthy subjects after RV infection (**Figure 2A**). However, publication bias assessment revealed that publication bias potentially existed, as indicated by Egger’s test (*p* < 0.05) and the presence of asymmetry of the Funnel plot (**Table 4** and **Supplementary Figure 10**). The largest effect size was obtained for IFN-λ (ES: -1.13) but it did not reach statistical significance (*p* = 0.230) (**Figure 2B**), probably due to a significant heterogeneity across studies (I^2^ = 89%, *p* < 0.001). As shown in **Table 5**, PBECs from children with atopic asthma also produced significantly lower levels of IFN-β (ES: -0.84, *p* = 0.030) (**Figure 3A**) and IFN-λ (ES: -1.00, *p* = 0.002) (**Figure 3B**). On the other hand, there were no significant differences in the levels of IL-6, IL-8, IP-10, and RANTES between PBECs isolated from adult asthmatic and healthy subjects (**Table 4** and **Supplementary Figures 1–4**), and the levels of IL-8 between PBECs isolated from children with asthma and healthy children (**Table 5** and **Supplementary Figure 5**), after RV infection.

**Table 4:** Summary of meta-analysis of *ex vivo* PBECs studies comparing RV-induced cytokine responses in adults with atopic asthma vs non-atopic healthy controls.

Cytokine	No. of Studies	Asthma/Healthy	Effect Size		Heterogeneity		Publication Bias				
			SMD (95% CI)	*p-value*	I^2^ Statistic	*p-value*	Egger’s Test (*p-value*)	Begg’s Test (*p-value*)	Rosenthal’s Failsafe-N	Trim and Fill (Number of Missing Studies)	Asymmetry of Funnel Plot
IL-6	4	49/41	-0.36	0.610	89%	<0.001	0.353	0.174	1	0	Absent
	(34, 39, 42, 45)		(-1.78, 1.05)								
IFN-β	4	36/34	-0.68	**0.009**	57%	0.070	0.014	0.174	21	0	Present
	(40, 43, 44)		(-1.20, -0.17)								
IL-8	3	31/27	-0.21	0.430	20%	0.290	0.283	0.602	0	0	Absent
	(34, 42, 45)		(-0.74, 0.31)								
IFN-λ	3	31/29	-1.13	0.230	89%	<0.001	0.225	0.117	10	0	Present
	(34, 44, 63)		(-2.98, 0.71)								
IP-10	3	40/32	-0.19	0.440	40%	0.190	0.205	0.602	0	0	Absent
	(34, 39, 45)		(-0.66, 0.29)								
RANTES	2	28/24	-0.51	0.080	56%	0.130	NA	NA	0	0	Absent
	(39, 45)		(-1.07, 0.05)								

CI, confidence interval; SMD, standardized mean difference; NA, not applicable; statistically significant results are in bold.

**Table 5:** Summary of meta-analysis of *ex vivo* PBECs studies comparing RV-induced cytokine responses in children with atopic asthma vs non-atopic healthy controls.

Cytokine	No. of Studies	Asthma/Healthy	Effect Size		Heterogeneity		Publication Bias				
			SMD (95% CI)	*p-value*	I^2^ Statistic	*p-value*	Egger’s Test (*p-value*)	Begg’s Test (*p-value*)	Rosenthal’s Failsafe-N	Trim and Fill (Number of Missing Studies)	Asymmetry of Funnel Plot
IL-8	2	19/20	-0.39	0.230	0%	0.570	NA	NA	0	0	Absent
	(22, 23)		(-1.03, 0.25)								
IFN-β	2	17/16	-0.84	**0.030**	37%	0.210	NA	NA	4	0	Absent
	(22, 23)		(-1.58, -0.10)								
IFN-λ	2	22/22	-1.00	**0.002**	0%	0.730	NA	NA	6	0	Absent
	(23)		(-1.63, -0.36)								

CI: confidence interval; SMD: standardized mean difference; NA: not applicable; statistically significant results are in bold.

#### Deficient IFN-γ Production in Atopic Asthmatic PBMCs After RV Infection

**Figure 4:**
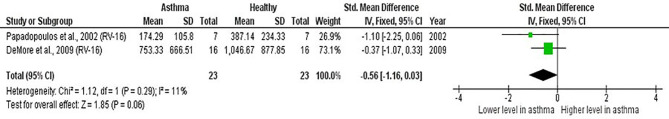
Forest plot of IFN-γ for *ex vivo* PBMCs studies comparing adults with atopic asthma vs non-atopic healthy controls. PBMCs from adults with atopic asthma produced lower levels of IFN-γ (ES: -0.56) compared with healthy adults after RV infection, and it was close to reaching statistical significance (*p* = 0.060).

Meta-analysis of PBMCs studies (**Table 6**) revealed that PBMCs from adults with atopic asthma produced lower levels of IFN-γ (ES: -0.56) compared with healthy adults, and it was close to reaching statistical significance (*p* = 0.060) (**Figure 4**). By contrast, the levels of IL-6, IL-10, IFN-α, and IFN-α2 in PBMCs of asthmatic and healthy subjects after RV infection were not significantly different (**Table 6** and **Supplementary Figures 6–9**). Publication bias was unclear as the low number of studies did not allow an accurate assessment of publication bias (e.g. Egger’s test and Begg’s test require a minimum of 3 studies).

**Table 6:** Summary of meta-analysis of *ex vivo* PBMCs studies comparing RV-induced cytokine responses in adults with atopic asthma vs non-atopic healthy controls.

Cytokine	No. of Studies	Asthma/Healthy	Effect Size		Heterogeneity		Publication Bias				
			SMD (95% CI)	*p-value*	I^2^ Statistic	*p-value*	Egger’s Test (*p-value*)	Begg’s Test (*p-value*)	Rosenthal’s Failsafe-N	Trim and Fill (Number of missing studies)	Asymmetry of Funnel Plot
IFN-α	3	56/52	-0.12	0.540	11%	0.330	0.471	0.602	0	0	Absent
	(44, 49)		(-0.50, 0.26)								
IFN-α2	2	38/36	-0.34	0.150	53%	0.140	NA	NA	0	0	Absent
	(47, 52)		(-0.81, 0.12)								
IFN-γ	2	23/23	-0.56	0.060	11%	0.290	NA	NA	2	0	Absent
	(47, 48)		(-1.16, 0.03)								
IL-6	2	28/28	-0.15	0.580	73%	0.060	NA	NA	0	0	Absent
	(47, 49)		(-0.69, 0.38)								
IL-10	2	23/23	0.09	0.750	20%	0.260	NA	NA	0	0	Absent
	(47, 48)		(-0.49, 0.68)								

CI, confidence interval; SMD, standardized mean difference; NA, not applicable; statistically significant results are in bold.

#### Lower Baseline IL-15 Levels in the Lower Respiratory Tract of Atopic Asthmatics Experimentally Inoculated With RV

**Figure 5:**
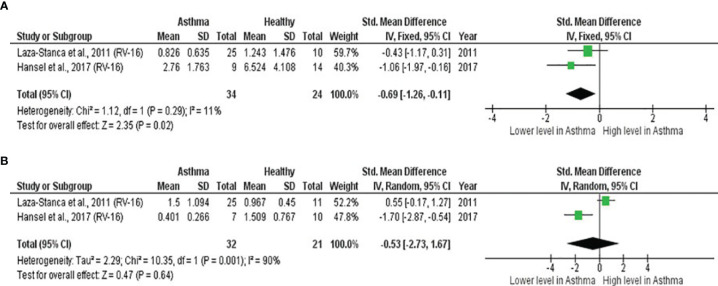
Forest plot of **(A)** baseline and **(B)** post-infection IL-15 for adult atopic asthmatics vs non-atopic healthy adults experimentally infected with RV. **(A)** Atopic asthmatics had significantly lower levels of bronchial IL-15 at baseline compared to non-atopic healthy individuals (ES: - 0.69, *p* = 0.020). **(B)** Atopic asthmatics also had lower levels of bronchial IL-15 following experimental inoculation with RV compared to non-atopic healthy individuals (ES: -0.53), but the difference was not statistically significant (*p* = 0.640).

**Figure 6:**
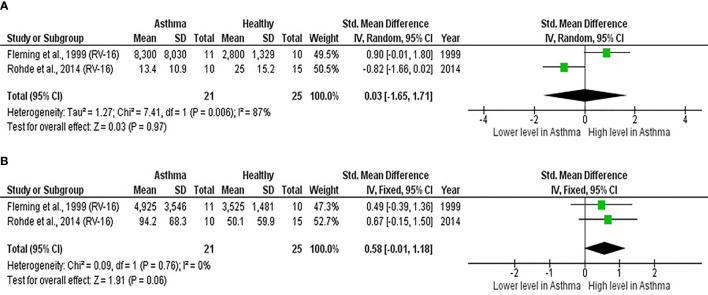
Forest plot of **(A)** baseline and **(B)** post-infection IL-8 for adult atopic asthmatics vs non-atopic healthy adults experimentally infected with RV. **(A)** At baseline, there was no significant difference in bronchial IL-8 levels between atopic asthmatics and non-atopic healthy adults. **(B)** After experimental RV infection, atopic asthmatics had higher levels of bronchial IL-8 (ES: 0.58) compared to non-atopic healthy individuals, and it was close to reaching statistical significance (*p* = 0.060).

As shown in **Table 7**, baseline bronchial (bronchial mucosal fluid and BAL) IL-15 levels were significantly lower in atopic asthmatics compared to non-atopic healthy individuals (ES: - 0.69, *p* = 0.020) (**Figure 5A**). IL-15 levels were also lower in atopic asthmatics following experimental inoculation with RV compared to non-atopic healthy individuals (ES: -0.53), but the levels were not statistically significant (*p* = 0.640) (**Figure 5B**). On the other hand, IL-8 levels detected in the lower respiratory tract (sputum and BAL) were higher in atopic asthmatics compared to non-atopic healthy individuals after experimental RV infection (ES: 0.58) with a *p-value* (0.060) close to reaching statistical significance (**Figure 6B**). At baseline (before experimental RV infection), there was no significant difference in bronchial IL-8 levels between asthma and healthy groups (**Figure 6A**). Publication bias was unclear due to the low number of studies (n = 2).

**Table 7:** Summary of meta-analysis of human experimental studies comparing bronchial cytokine responses in adults with atopic asthma vs non-atopic healthy controls before (baseline) and after (post-infection) experimental RV infection.

Cytokine	No. of Studies	Asthma/Healthy	Effect Size		Heterogeneity		Publication Bias				
			SMD (95% CI)	*p-value*	I^2^ Statistic	*p-value*	Egger’s Test (*p-value*)	Begg’s Test (*p-value*)	Rosenthal’s Failsafe-N	Trim and Fill (Number of missing studies)	Asymmetry of Funnel Plot
IL-15 (Baseline)	2	34/24	-0.69	**0.020**	11%	0.290	NA	NA	3	0	Absent
	(54, 59)		(-1.26, -0.11)								
IL-15 (Post-infection)	2	32/21	-0.53	0.640	90%	0.001	NA	NA	0	0	Absent
	(54, 59)		(-2.73, 1.67)								
IL-8 (Baseline)	2	21/25	0.03	0.970	87%	0.006	NA	NA	0	0	Absent
	(56, 58)		(-1.65, 1.71)								
IL-8 (Post-infection)	2	21/25	0.58	0.060	0%	0.760	NA	NA	1	0	Absent
	(56, 58)		(-0.01, 1.18)								

CI, confidence interval; SMD, standardized mean difference; NA, not applicable; statistically significant results are in bold.

## Discussion

RV accounts for most virus-induced asthma exacerbations, and existing literature suggests that the clinical illness severity is potentially linked to host immune responses, including the epithelial-derived cytokines and cytokines/chemokines secreted by the immune cells. However, there were large variations between previous reports on RV-induced cytokine responses. Therefore, the present study aimed to identify cytokines/chemokines that play an essential role in the pathogenesis of RV-induced asthma exacerbations and determine whether differential cytokine responses to RV infection exist between asthmatic and healthy individuals.

Although RV infections have been regarded as a major cause of asthma exacerbations, much scientific evidence on RV-induced pathological changes in asthma were obtained from observational studies. The major drawback of these studies is that the causal link between RV infection and the clinical findings in human subjects could not be established. Furthermore, the early host responses before symptoms manifestation could not be determined (64). Thus, the best study design to explore the pathophysiological mechanisms of RV-induced asthma exacerbations involves inoculation of human volunteers with RV followed by sampling biological specimens such as nasal lavage/fluid, bronchial fluid, BAL, and sputum at different time points. This allows for the elucidation of inflammatory changes in both the upper (nasal) and lower (bronchial) airways during the period of RV infection (64). However, RV inoculation did not always lead to more severe symptoms in asthmatic individuals (56). Furthermore, in some of these human experimental studies, no correlation analysis was done, or no correlation was found between the levels of cytokines and respiratory symptoms (57, 58). Among asthmatic subjects with significantly increased respiratory symptoms following RV inoculation, type 2 inflammation is a significant factor contributing to RV-induced asthma exacerbations (54, 55). Experimental RV infection of asthmatic subjects resulted in increased IL-4, IL-5, and IL-13 levels. The nasal and bronchial IL-5 and IL-13 levels were also positively correlated with the severity of upper and lower respiratory symptoms (54, 55). Evidence suggests that IL-25 and IL-33 are key cytokines driving the type 2 inflammatory responses in asthmatic airways (46, 55). Besides that, a meta-analysis of bronchial cytokine levels reveals that baseline IL-15 levels were significantly lower in asthmatics compared to healthy individuals (**Table 7**, **Figure 5A**), suggesting that the difference in lower airway microenvironment before exposure to RV infection might influence the severity of asthma exacerbations. Although IL-4, IL-5, and IL-13 are commonly implicated in the pathogenesis of allergic asthma, IL-6 and IL-8 may play an important role in regulating the immune responses to RV infection as elevated levels of IL-6 and IL-8 were frequently found in the upper as well as lower airways (47, 56, 58, 60). Evidence has shown that IL-6 can stimulate IL-4 secretion and promote Th2 and/or Th17 differentiation (65), whereas IL-8 is a well-known neutrophil chemoattractant that may promote neutrophilic inflammation in asthmatic airways (66).

Airway epithelial cells are the primary target of RV in the respiratory tract. As RV-induced colds are generally mild and self-limiting, RV-induced asthma exacerbations are believed to be caused by an ineffective antiviral defence mechanism in airway epithelium that leads to delayed virus clearance. In fact, earlier studies showed that airway epithelial cells of asthmatics failed to produce similar levels of IFNs such as IFN-β and IFN-λ compared to that of healthy individuals when infected with RV *ex vivo* (33, 40, 43). Asthmatic PBECs were also demonstrated to have increased susceptibility to RV infection due to deficient IFNs production as a higher viral load and a more significant amount of infectious virus particles were detected compared to healthy PBECs (40). Apart from PBECs, impaired immune responses against RV infection have also been reported in PBMCs and BAL cells (mainly alveolar macrophages), particularly for IFN-α and IFN-γ (48, 51). However, more recent studies could not demonstrate such differences in IFNs production in PBECs (22, 24, 42) and PBMCs (44, 47) and thus raised questions on the role of IFNs in asthma exacerbations. Nevertheless, a meta-analysis conducted in this study confirmed that PBECs from adults with atopic asthma had impaired production of IFN-β. By contrast, children with atopic asthma had impaired production of IFN-β and IFN-λ in response to RV infection compared to non-atopic healthy controls. Besides that, there was a trend towards a deficient production of IFN-γ in PBMCs from adult asthmatics compared to healthy subjects. The comparison of cytokine responses in this meta-analysis is limited to atopic asthmatics and non-atopic healthy individuals to eliminate any potential influence on RV-induced cytokine responses due to the atopic status.

The reduced ability of asthmatic airway epithelial cells to secrete IFNs could profoundly affect the pathogenesis of RV infection in people with asthma, leading to an increased risk of asthma exacerbations. There are three classes of IFNs, which are type I (IFN-α and IFN-β), type II (IFN-γ), and type III (IFN-λ) (67). Type I and III IFNs have direct antiviral effects of limiting virus replication by inducing an antiviral state in virus-infected and bystander cells through the activation of IFN-stimulated genes (ISGs) (68, 69). On the other hand, type II IFN, or IFN-γ, is mainly produced by natural killer cells in response to RV infection as an innate defence mechanism (70). IFN-γ is also primarily produced by CD4 T cells with Th1 phenotype (71), thus a deficient IFN-γ production in PBMCs from the asthmatic individuals might reflect that asthmatic individuals have a lower percentage of Th1 cells. Although we did not find significant differences in the levels of other proinflammatory cytokines and chemokines produced by RV-infected PBECs and PBMCs between asthmatic and healthy individuals, the intrinsic differences in their capability to produce certain cytokines such as IFNs could potentially influence the ultimate outcome in the airway milieu. Given the immunomodulatory role of IFNs in promoting the Th1 phenotype of CD4 T cells (68), the defective innate immune responses in airway epithelium – the primary site of RV infection and replication – might lead to a skewed Th2 response and an enhanced type 2 inflammation in asthmatic airways. This may explain why RV infection could cause more detrimental effects in asthmatics’ lower respiratory tract than healthy individuals.

While this systematic review/meta-analysis described the differences in the levels of cytokines between atopic asthmatic and healthy individuals at cellular levels and in the respiratory tracts, there are several factors, other than the atopic status of the subjects, that might influence RV-induced cytokine responses. Since the publication period for the included studies ranges from 2002 to 2021, the detection methods used to measure the levels of cytokines, including ELISA and other immunoassays, and their sensitivity varied (**Supplementary Table 4–6**). Thus, the detection limits of these assays might be a potential confounding factor that influences the results, especially for lowly expressed cytokines. In some studies discussed here, IFN-β and IFN-λ were not detected in the PBEC cultures of asthmatic and healthy individuals (22, 27, 35). Besides that, most of the included studies, particularly *ex vivo* studies, measured the levels of cytokines for both asthma and healthy groups at the same time points, and the kinetics of cytokine induction was not investigated. A recent study by Veerati et al. (72) has shown that IFNs induction in PBECs from asthmatic and COPD donors was delayed rather than deficient than PBECs from healthy donors. However, since the asthmatic subjects in the study by Veerati et al. (72) comprised both atopic and non-atopic individuals, it remains unclear whether similar results will be obtained if only atopic asthmatics were recruited. It is also important to note that, despite a delayed induction of IFNs, IFN-λ protein levels were significantly lower in asthmatic PBECs than healthy PBECs at a later time-point, i.e. 96hr post-infection. By contrast, no significant difference was observed for IFN-β protein levels (72). As asthma is increasingly recognised as a heterogeneous disease, the dysregulated cytokine responses may be linked to specific molecular phenotypes in asthmatic individuals. A transcriptomics study by Khoo et al. (73) demonstrated that, for children presented to the emergency department due to wheezing or acute exacerbation of asthma, IRF7^lo^ molecular phenotype, which was characterized by up-regulation of cytokine and growth factor signalling and down-regulation of IFN-γ, was associated with a higher chance of hospital admission and recurrence compared to IRF7^hi^, which was accompanied by a strong Th1/IFN response. While more studies are required to uncover the molecular phenotypes or endotypes of asthma associated with increased susceptibility to RV infection, some studies suggest that high serum IgE levels in atopic asthma increase the risk of RV-induced asthma exacerbations. A study by Zambrano et al. (74) has shown that experimental RV infection caused more significant lower respiratory symptoms in young atopic adults with high levels of total serum IgE than those with low levels of total serum IgE. Consistent with this, administering omalizumab, an anti-IgE antibody, has been demonstrated to reduce lower respiratory tract symptoms and improve lung function in atopic asthmatics challenged with RV (64).

As discussed above, defective innate immune responses at cellular levels may contribute to an imbalance in Th1/Th2 cytokines in asthmatic airways. In view of the finding that the severity of RV-induced asthma exacerbations is potentially linked to an imbalance in Th1/Th2 cytokines, exogenous IFNs may offer great therapeutic benefits owing to their antiviral and immunomodulatory activities. Type I IFNs such as IFN-α and IFN-β are antiviral cytokines that had been tested for their efficacy against RV infection in clinical trials. Intranasal IFN-α2 treatment, however, was shown to be ineffective against natural occurring colds caused by RV infection (75). On the other hand, a clinical trial demonstrated that inhaled IFN-β had no significant effect on the asthma symptoms because viral infections did not cause significant deteriorations of asthma among the recruited patients. However, it was found to reduce asthma symptoms in a subset of patients with difficult-to-treat asthma (76). Given the critical role of IFN-λ in innate and adaptive immune responses (69), it is also worthwhile to evaluate the effectiveness of IFN-λ treatment in preventing RV- or virus-induced asthma exacerbations. Currently, IFN-λ is being assessed for its efficacy against chronic hepatitis C virus infection and coronavirus disease 2019 (COVID-19) in clinical trials (77, 78). Exogenous IFN-β and IFN-λ may have prophylactic effects against RV-induced asthma exacerbations as there is increasing evidence showing that the currently used drugs for asthma management such as inhaled corticosteroids (ICS) and long-acting-β2 agonists (LABA) can diminish the antiviral immunity against RV infection (49, 79).

A few limitations of this systematic review/meta-analysis should be noted. Firstly, the meta-analysis is limited by the number of studies with the same experimental design, resulting in a few studies investigating the same cytokine with relatively small sample size. Thus, additional comparisons/subgroup analyses for different asthma severity or RV serotypes cannot be performed. Besides that, the human experimental studies included in the systematic review/meta-analysis are limited to RV-16 only due to safety and ethical concerns. RV-16 is specifically used to inoculate human volunteers because it is a relatively safer RV serotype and known to not cause very severe exacerbations in asthmatic patients (80). The immune responses and the clinical outcomes observed in human subjects might differ when natural infections with other RV serotypes occur. Considering that there are other more pathogenic serotypes of RV, the differences in cytokine responses and clinical outcomes might be more contrasting between asthmatic and healthy individuals, and this should be further investigated in future studies. While some epidemiological studies suggest that RV-C is more likely to be associated with pneumonia (81) and wheezing (82), as well as severe asthma (83) compared to RV-A and RV-B, there is no study that compares RV-C-induced cytokine responses between asthmatic and healthy individuals yet. A study by Nakagome et al. (84) has shown that RV-A and RV-C replicated more rapidly and induced higher cytokine levels than RV-B in differentiated human sinus epithelial cells (HSECs), suggesting that RV-induced cytokine responses may be species-dependent. Thus, it would be interesting to investigate whether RV-C strains induce different levels of cytokines in cells originating from asthmatic and healthy individuals. As RV-C targets ciliated airway epithelial cells and can be propagated in differentiated airway epithelial cells (85), a comparison of cytokine levels between PBECs from asthmatic and healthy individuals could be carried out after the cells undergo differentiation at the air-liquid interface.

To identify the differences between asthmatic and healthy individuals before and/or after RV infection, which may contribute to RV-induced asthma exacerbations, the present study discusses three commonly used experimental models (PBECs, PBMCs, and human experimental studies). Apart from that, other relevant experimental models, such as human airway explant, may improve our understanding of the pathophysiological mechanisms of RV-induced asthma exacerbations. Using human airways precision lung cut slices (PCLS), Kennedy et al. (86) has shown that RV-39 infection resulted in a more significant constriction in asthmatic airways than non-asthmatic airways in response to carbachol. Asthmatic lung explants were also found to have an enhanced gene expression of IL-25, thymic stromal lymphopoietin (TSLP), and IL-13 compared to non-asthmatic tissue, suggesting that airway hyperresponsiveness may be related to these cytokines (86). Besides that, recent evidence suggests that B cells may also play an important role in the systemic responses to RV infection. A study by Wirz et al. (87) demonstrated that experimental RV infection could induce antiviral responses in peripheral B cells, and these responses were more broadly elevated and dysregulated in asthma patients. While *in vitro* studies on single-cell types revealed many intrinsic differences between asthmatic and healthy individuals, they may not represent the conditions during RV-induced asthma exacerbations due to the absence of cell-cell interactions. On the other hand, human experimental studies allow the assessment of host responses *in vivo*. However, as inoculation of human volunteers is restricted to a particular RV serotype (RV-16), findings from community-based studies should also be considered to elucidate the pathophysiological mechanisms of RV-induced asthma exacerbations, especially for virulent RV strains such as those of RV-C species.

## Conclusion

RV-induced asthma exacerbations are potentially caused by enhanced Th2 responses (IL-4, IL-5, and IL-13), which could be promoted by IL-25 and IL-33, and IL-15 deficiency before RV infection. The imbalance between Th1 and Th2 cytokines in the airways during RV-induced asthma exacerbations may be partly contributed by defective innate immune responses in airway epithelial cells and peripheral blood mononuclear cells. Through meta-analysis, we further confirmed that asthmatic airway epithelial cells have defective IFNs (IFN-β and IFN-λ) production, and these changes could occur in both adults and children with atopic asthma. Exogenous IFNs delivery may be beneficial as a prophylactic approach to prevent RV-induced asthma exacerbations.

## Data Availability Statement

The original contributions presented in the study are included in the article/**Supplementary Material**. Further inquiries can be directed to the corresponding author.

## Author Contributions

KYL, SKK, SLH, and MKLN performed database searching, article screening, study selection and appraisal, and data extraction and analysis. KYL and CLT conceived the idea and wrote the manuscript. CLT supervised the study and resolved conflicts between reviewers (KYL, SKK, SLH, and MKLN). H-YC, HHH, and DAI provided important information for the completion of the manuscript. All authors read and approved the final manuscript.

## Funding

This study was funded by Fundamental Research Grant Scheme (FRGS/1/2018/SKK10/UPM/02/2) under the Ministry of Higher Education Malaysia. KYL was funded by Universiti Putra Malaysia Graduate Research Assistantship (GRA) and Special Graduate Research Allowance Scheme (SGRA).

## Conflict of Interest

The authors declare that the research was conducted in the absence of any commercial or financial relationships that could be construed as a potential conflict of interest.

## Publisher’s Note

All claims expressed in this article are solely those of the authors and do not necessarily represent those of their affiliated organizations, or those of the publisher, the editors and the reviewers. Any product that may be evaluated in this article, or claim that may be made by its manufacturer, is not guaranteed or endorsed by the publisher.
