# Supplementary material for: Rhinovirus-Induced Cytokine Alterations With Potential Implications in Asthma Exacerbations: A Systematic Review and Meta-Analysis
Source: Front Immunol. 2022 Feb 15;13:782936. doi: 10.3389/fimmu.2022.782936 (PMC8886024; doi:10.3389/fimmu.2022.782936)
Supplement: Supplementary file 1 [file DataSheet_1.docx]

Supplementary Material

# Supplementary Figures and Tables

## Supplementary Figures


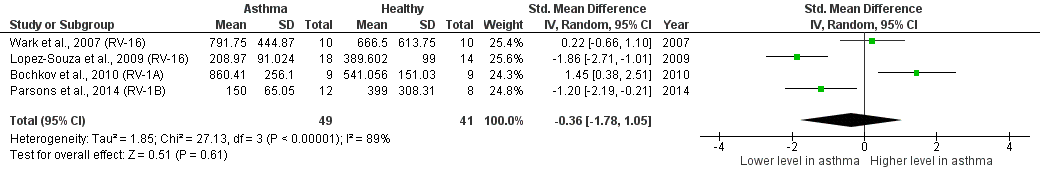


**Supplementary Figure 1.** Forest plot of IL-6 for *ex vivo* PBECs studies comparing adults with atopic asthma vs non-atopic healthy controls.


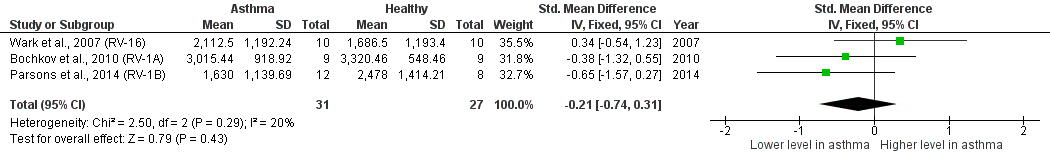


**Supplementary Figure 2.** Forest plot of IL-8 for *ex vivo* PBECs studies comparing adults with atopic asthma vs non-atopic healthy controls.


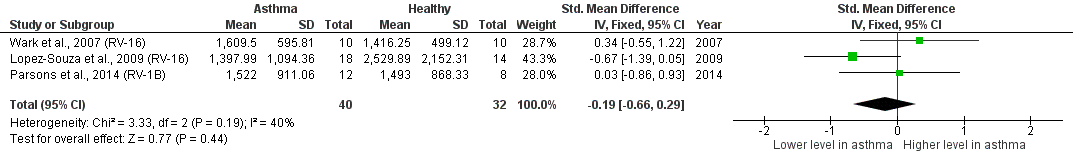


**Supplementary Figure 3.** Forest plot of IP-10 for *ex vivo* PBECs studies comparing adults with atopic asthma vs non-atopic healthy controls.


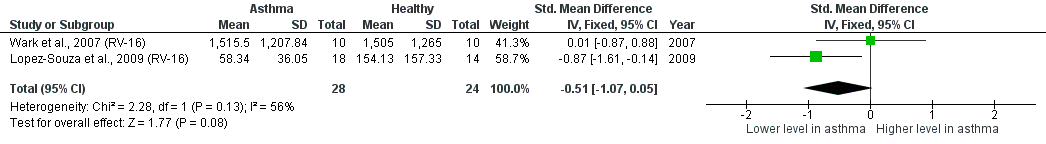


**Supplementary Figure 4.** Forest plot of RANTES for *ex vivo* PBECs studies comparing adults with atopic asthma vs non-atopic healthy controls.


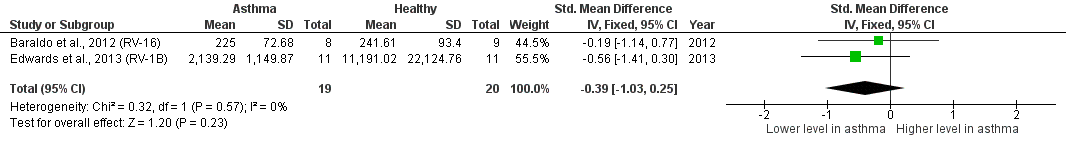


**Supplementary Figure 5.** Forest plot of IL-8 for *ex vivo* PBECs studies comparing children with atopic asthma vs non-atopic healthy controls.


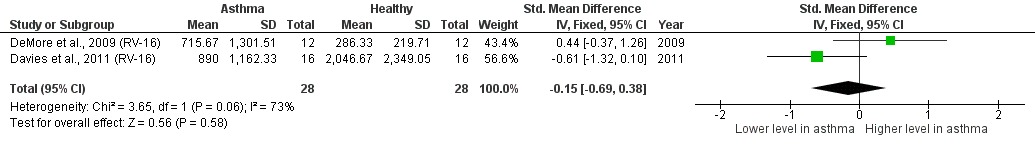


**Supplementary Figure 6.** Forest plot of IL-6 for *ex vivo* PBMCs studies comparing adults with atopic asthma vs non-atopic healthy controls.


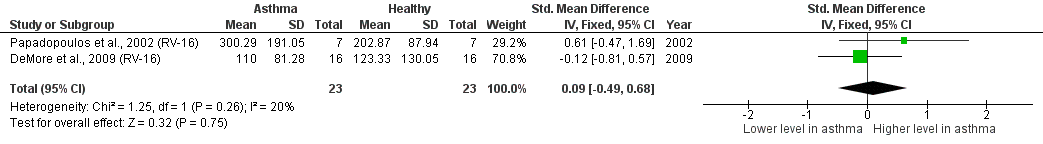


**Supplementary Figure 7.** Forest plot of IL-10 for *ex vivo* PBMCs studies comparing adults with atopic asthma vs non-atopic healthy controls.


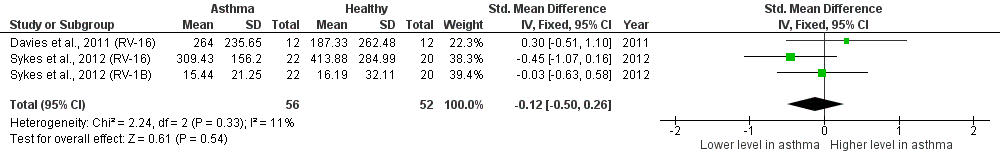


**Supplementary Figure 8.** Forest plot of IFN-α for *ex vivo* PBMCs studies comparing adults with atopic asthma vs non-atopic healthy controls.


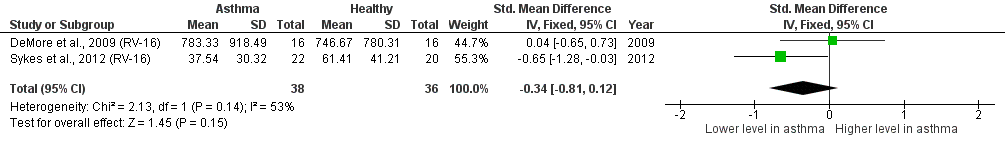


**Supplementary Figure 9.** Forest plot of IFN-α2 for *ex vivo* PBMCs studies comparing adults with atopic asthma vs non-atopic healthy controls.


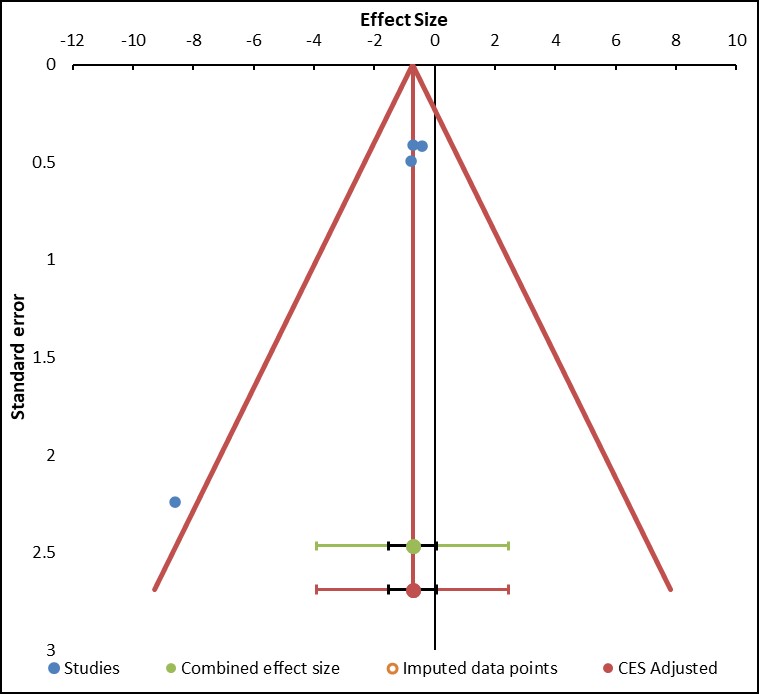


**Supplementary Figure 10.** Funnel plot of IFN-β for *ex vivo* PBECs studies comparing adults with atopic asthma vs non-atopic healthy controls.

## Supplementary Tables

**Supplementary Table 1.** Subject characteristics of *ex vivo* PBECs studies comparing RV-induced cytokine responses in asthmatic vs healthy individuals.

| **Study** | **Asthmatics** | | | | | | | **Healthy Controls** | | | | |
| --- | --- | --- | --- | --- | --- | --- | --- | --- | --- | --- | --- | --- |
|  | **n** | **Age** | **Male / Female** | **Allergy Status** | **Asthma Severity** | **ICS Intake** | **Smoker (Y/N)** | **n** | **Age** | **Male / Female** | **Allergy Status** | **Smoker (Y/N)** |
| Wark et al., 2005 | 10 (Mild)  14 (Moderate) | 32 (12.6)  32 (21-58) | 6/4  10/4 | Atopic | Mild, moderate | Mild – No ICS, Moderate – Taking ICS | No | 10 | 29 (24-38) | 6/4 | Non-atopic | No |
| Contoli et al., 2006 | 9 | 31.2 (21-50) | 5/4 | Atopic | Mild, moderate, severe | 7 out of 9 taking ICS | No | 9 | 27.8 (23-31) | 6/3 | Non-atopic | No |
| Wark et al., 2007 | 10 | 31 (12.6) | 4/6 | Atopic | Mild | No ICS | No | 10 | 30 (6.9) | 4/6 | Non-atopic | No |
| Wark et al., 2009 | 4 | 36.5 (28-44) | 2/2 | Atopic | Mild | Taking ICS | No | 4 | 38.75 (28-50) | 2/2 | Non-atopic | No |
| Lopez-Souza et al., 2009 | 6 | 33 (24-50) | 4/2 | Atopic | Mild-to-moderate | No ICS | No | 5 | 27 (32-46) | 3/2 | Non-atopic | No |
| Bochkov et al., 2010 | 9 | 25.4 (19-35) | 4/5 | Atopic | Mild | NS | NS | 9 | 25.2 (19-41) | 3/6 | Non-atopic | NS |
| Cakebread et al., 2011 | 10 | 37 (23-70) | 4/6 | NS | NS | 6 taking ICS | NS | 6 | 29 (20-44) | 1/5 | NS | NS |
| Baraldo et al., 2012 | 8 (Atopic)  9 (Non-atopic) | 5 (2-13)  NS | 6/2  NS | Atopic  Non-atopic | NS | 3 taking ICS | No | 9  9 | 5 (2-7)  NS | 4/5  NS | Non-atopic  Atopic | No |
| Bedke et al., 2012 | 35 | 40.2 (19-70) | 14/21 | NS | NS | 25 taking ICS | NS | 24 | 27.2 (20-55) | 9/15 | NS | NS |
| Edwards et al., 2013 | 11 | 11 (9-15) | 6/5 | Atopic | Severe | Taking ICS | No | 11 | 7 (2-15) | 7/4 | Non-atopic | No |
| Sykes et al., 2014 | 10 | 34.2 (8.4) | 8/2 | Atopic | Mild, moderate | Half taking ICS | No | 12 | 38.5 (11.1) | 5/7 | Non-atopic | No |
| Parsons et al., 2014 | 12 | 54.5 (9.9) | 3/9 | Atopic | Moderate-to-severe | Taking ICS | No | 8 | 55.9 (12.3) | 4/4 | Non-atopic | No |
| Bochkov et al., 2013 | 8 | 30.75 (20-52) | 4/4 | Atopic | Moderate-to-severe | Taking ICS | NS | 9 | 24.78 (20-40) | 3/6 | Non-atopic | NS |
| Beale et al., 2014 | 10 | 34.5 (2.3) | 7/3 | Atopic | Moderate | 8 taking ICS | NS | 10 | 28 (2.4) | 3/7 | Non-atopic | NS |
| Bai et al., 2015 | 6 | 46.3 (9-64) | 0/6 | NS | Severe | Taking ICS | 3 smokers | 6 | 41.7 (13-62) | 4/2 | NS | 2 smokers |
| Kicic et al., 2016 | 39 | 8.2 (2.6-14.8) | 25/14 | Atopic | Mild | No ICS | No | 36 | 8.4 (3.2-15.6) | 18/18 | Non-atopic | No |
| Moskwa et al., 2018 | 6 (Atopic)  4 (Non  -atopic) | 29 (6.1)  40.25 (10.69) | 5/1  2/2 | Atopic  Non-atopic | Mild, severe | Taking ICS | NS | 9 | 31.78 (14.62) | 5/4 | Non-atopic | NS |
| Williams et al., 2021 | 8 | 61.5 (13.6) | 1/7 | Atopic and non-atopic | Moderate, severe | Taking ICS | NS | 8 | 61.5 (8.8) | 3/5 | Atopic and non-atopic | NS |

**Supplementary Table 2.** Subject characteristics of *ex vivo* PBMCs studies comparing RV-induced cytokine responses in asthmatic vs healthy individuals.

| **Study** | **Asthmatics** | | | | | | | **Healthy Controls** | | | | |
| --- | --- | --- | --- | --- | --- | --- | --- | --- | --- | --- | --- | --- |
|  | **n** | **Age** | **Male / Female** | **Allergy Status** | **Asthma Severity** | **ICS Intake** | **Smoker (Y/N)** | **n** | **Age** | **Male / Female** | **Allergy Status** | **Smoker (Y/N)** |
| Papadopoulos et al., 2002 | 7 | 20-57 | 3/4 | Atopic | Mild-to-moderate | 2 taking ICS | NS | 7 | 20-57 | 3/4 | Non-atopic | NS |
| Xatzipsalti et al., 2007 | 12 | 30 (19-40) | 8/4 | Atopic | NS | No ICS | No | 12 | 30 (19-40) | 8/4 | Non-atopic | No |
| DeMore et al., 2009 | 16 | NS | NS | Atopic | Mild | No ICS | No | 16 | NS | NS | Non-atopic | No |
| Davies et al., 2011 | 12 | 30 (28.33-40.91) | 0/12 | Atopic | Mild-to-moderate | 6 taking ICS | NS | 12 | 43.46 (33.54-50.81) | 1/11 | Non-atopic | NS |
| Iikura et al., 2011 | 39 | 19 (7-35) | 21/18 | Atopic and non-atopic | Mild, moderate | Taking ICS | No | 50 | 19 (7-35) | 29/21 | Atopic and non-atopic | No |
| Sykes et al., 2012 | 22 | 33.3 (1.713) | 16/6 | Atopic | Mild | 63% taking ICS | No | 20 | 38.4 (2.426) | 9/11 | Non-atopic | No |
| Pritchard et al., 2014 | 22 | 33.83 (12.9) | 11/11 | Atopic | Mild-to-moderate | 12 taking ICS | No | 20 | 35.3 (12.6) | 10/10 | Non-atopic | No |
| Upton et al., 2017 | 15 | NS | NS | Atopic | Mild | NS | NS | 15 | NS | NS | Non-atopic | NS |
| Jurak et al., 2018 | 18 | 18-53 | NS | Atopic and non-atopic | Mild, moderate | Taking ICS | No | 22 | 18-53 | NS | Atopic and non-atopic | No |
| Hosseini et al., 2021 | 48 | 5.25 (3.82-6.99) | 35/13 | Atopic and non-atopic | NS | 33 taking ICS | No | 14 | 6.58 (4.99, 8.38) | NS | Non-atopic | No |

**Supplementary Table 3.** Subject characteristics of human experimental studies comparing RV-induced cytokine responses in asthmatic vs healthy individuals.

| **Study** | **Asthmatics** | | | | | | | **Healthy Controls** | | | | |
| --- | --- | --- | --- | --- | --- | --- | --- | --- | --- | --- | --- | --- |
|  | **n** | **Age** | **Male / Female** | **Allergy Status** | **Asthma Severity** | **ICS Intake** | **Smoker (Y/N)** | **n** | **Age** | **Male / Female** | **Allergy Status** | **Smoker (Y/N)** |
| Fleming et al., 1999 | 11 | 34.7 (24-56) | 5/6 | Atopic | Mild | No ICS | No | 10 | 29.3 (19-43) | 2/8 | Non-atopic | No |
| Kluijver et al., 2003 | 25 | 22.28 (19-25) | 12/13 | Atopic | Mild-to-moderate | 12 taking ICS | NS | 12 | 21.5 (19-27) | 9/3 | Non-atopic | NS |
| DeMore et al., 2009 | 20 | 22.3 (4.1) | 7/13 | Atopic | Mild | No ICS | No | 17 | 24.2 (7.3) | 5/12 | Non-atopic | No |
| Laza-Stanca et al., 2011 | 10 | 22.9 (19-31) | 2/8 | Atopic | Mild | No ICS | No | 15 | 26.9 (18-53) | 8/7 | Non-atopic | No |
| Rohde et al., 2014 | 10 | 22.9 (19-31) | 2/8 | Atopic | Mild | No ICS | No | 15 | 26.9 (18-53) | 8/7 | Non-atopic | No |
| Beale et al., 2014 | 11 (Mild)  17 (Moderate) | 33 (11)  37 (10) | 4/7  9/8 | Atopic | Mild, moderate | Mild – No ICS, Moderate – 15 taking ICS | No | 11 | 31 (12) | 7/4 | Non-atopic | No |
| Jackson et al., 2014 | 28 | 36 (11) | 13/15 | Atopic | Mild, moderate | 15 taking ICS | No | 11 | 31 (12) | 7/4 | Non-atopic | No |
| Upton et al., 2017 | 11 (Mild)  14 (Moderate) | NS | NS | Atopic | Mild, moderate | Taking ICS | No | 10 | NS | NS | Non-atopic | No |
| Hansel et al., 2017 | 28 | 36 (11) | 13/15 | Atopic | Mild, moderate | 15 taking ICS | No | 11 | 31 (12) | 7/4 | Non-atopic | No |
| Williams et al., 2021 | 28 | 36 (11) | 13/15 | Atopic | Mild, moderate | 15 taking ICS | No | 11 | 31 (12) | 7/4 | Non-atopic | No |

**Supplementary Table 4.** Experimental details of *ex vivo* PBECs studies comparing RV-induced cytokine responses in asthmatic vs healthy individuals.

| **Study** | **Study Population** | **RV Serotype (Dose)** | **Sample Type** | **Detection Method** | **Measured Cytokines** | **Sensitivity / Detection Limit** |
| --- | --- | --- | --- | --- | --- | --- |
| Wark et al., 2005 | Adult | RV-16, RV-1B (2 MOI) | Culture supernatant | ELISA | IL-6, RANTES, IFN-β | IL-6 (3 pg/mL)  RANTES (2 pg/mL)  IFN-β (250 pg/mL) |
| Contoli et al., 2006 | Adult | RV-16 (2 MOI) | Culture supernatant | ELISA | IFN-λ1 | IFN-λ1 (25 pg/mL)  (5 and 25% cross reactivity with IFN-λ2/3) |
| Wark et al., 2007 | Adult | RV-16 (2 MOI) | Culture supernatant | ELISA & CBA | IL-6, IL-8, IP-10, RANTES, TNF-α | IL-6 (CBA: 2.5 pg/mL; ELISA: 3 pg/mL)  IL-8 (CBA: 0.2 pg/mL; ELISA: 5 pg/mL)  IP-10 (CBA: 2.8 pg/mL)  RANTES (CBA: 1 pg/mL)  TNF-α (CBA: 3.7 pg/mL; ELISA: 0.09 pg/mL) |
| Wark et al., 2009 | Adult | RV-1B (1 MOI) | Culture supernatant | ELISA | IFN-β | IFN-β (25 pg/mL) |
| Lopez-Souza et al., 2009 | Adult | RV-16 (1 MOI) | Culture supernatant | LINCOplex human cytokine kit | IL-1α, IL-6, IP-10, RANTES | NS |
| Bochkov et al., 2010 | Adult | RV-1A (10 MOI) | Culture supernatant | ELISA & Beadmates assay | IL-1β, IL-6, IL-8 | IL-1β, IL-6 (8.2 pg/mL)  IL-8 (NS) |
| Cakebread et al., 2011 | Adult | RV-1B (1 x 10^3^ TCID_50_/10^5^ cells) | Culture supernatant | ELISA & CBA | IL-6, IL-8, IP-10, RANTES | NS |
| Baraldo et al., 2012 | Children | RV-16 (5 MOI) | Culture supernatant | ELISA | IL-8, IFN-β, IFN-λ | IL-8 (31.2 pg/mL)  IFN-β (25 pg/mL)  IFN-λ (20 pg/mL) |
| Bedke et al., 2012 | Adult | RV-1B (1000 or 5000 TCID_50_/10^5^ cells) | Culture supernatant | ELISA | TGF-β1, TGF-β2 | NS |
| Edwards et al., 2013 | Children | RV-16, RV-1B (2 MOI) | Culture supernatant | ELISA | IL-8, RANTES, IFN-β, IFN-λ | IL-8 (15 pg/mL)  IFN-β (1 pg/mL)  IFN-λ (15 pg/mL)  RANTES (NS) |
| Sykes et al., 2014 | Adult | RV-16, RV-1B (1 MOI) | Culture supernatant | ELISA | IFN-α, IFN-β, IFN-λ | All 10 pg/mL |
| Parsons et al., 2014 | Adult | RV-1B (20 MOI) | Culture supernatant | ELISA & CBA | IL-6, IL-8, IP-10, IFN-λ1/3 | NS |
| Bochkov et al., 2013 | Adult | RV-16 (5 x 10^6^ PFU/mL) | Culture supernatant | ELISA & Milliplex map kits | IL-8, IP-10, TNF-α, IFN-β, IFN-λ2/3 | IL-8 (0.3 pg/mL)  IP-10 (2.2 pg/mL)  TNF-α (0.1 pg/mL)  IFN-β (50 pg/mL)  IFN-λ2/3 (7.9 pg/mL) |
| Beale et al., 2014 | Adult | RV-1B (2 MOI) | Culture supernatant | ELISA | IL-25 | NS |
| Bai et al., 2015 | Adult | RV-16 (5 x 10^6^ PFU in 50uL medium) | Culture supernatant | Luminex 42-plex assay | IL-1α, IL-1β IL-1Ra, IL-2, IL-3, IL-4 IL-5, IL-6, IL-7, IL-8, IL-9, IL-10, IL-12p40, IL-12p70, IL-13, IL-15, IL-17, GRO, G-CSF, GM-CSF, fractalkine, FIT3L, eotaxin, IFN-α, IFN-γ, IP-10, MCP-1, MCP-3, MDC, MIP-1α, MIP-1β, PDGRA, PDG-BB, EGF, FGF2, VEGF, RANTES, sCD40L, sIL2-Ra, TGF-α, TNF-α, TNF-β | NS |
| Kicic et al., 2016 | Children | RV-1B, RV-14 (5 x 10^5^ TCID_50_/mL) | Culture supernatant | ELISA & DELFIA | IL-1β, IL-6, IL-8, IP-10, RANTES, IFN-α, IFN-β, IFN-λ | NS |
| Moskwa et al., 2018 | Adult | RV-1B (0.1 MOI) | Culture supernatant | ELISA & CBA | IP-10, RANTES, IFN-α, IFN-β, IFN-λ1 | IP-10 (10 pg/mL)  RANTES (10 pg/mL)  IFN-α (10 pg/mL)  IFN-β (50 pg/mL)  IFN-λ1 (15.6 pg/mL) |
| Williams et al., 2021 | Adult | RV-1A (0.1 MOI) | Culture supernatant | ELISA | MDC, TARC | NS |

**Supplementary Table 5.** Experimental details of *ex vivo* PBMCs studies comparing RV-induced cytokine responses in asthmatic vs healthy individuals.

| **Study** | **Study Population** | **RV Serotype (Dose)** | **Sample Type** | **Detection Method** | **Measured Cytokines** | **Sensitivity / Detection Limit** |
| --- | --- | --- | --- | --- | --- | --- |
| Papadopoulos et al., 2002 | Adult | RV-16 (1 MOI) | Culture supernatant | ELISA | IL-4, IL-5, IL-10, IL-12, IL-13, IFN-γ | IL-4 (0.27 pg/mL)  IL-5 (4 pg/mL)  IL-10 (7 pg/mL)  IL-12 (7 pg /mL)  IL-13 (12 pg/mL)  IFN-γ (6 pg/mL) |
| Xatzipsalti et al., 2007 | Adult | RV-16, RV-1B (5 MOI) | Culture supernatant | ELISA | IL-6, IL-8, IL-10, RANTES, IFN-γ, TGF-β1 | NS |
| DeMore et al., 2009 | Adult | RV-16 (1 x 10^7^ PFU/mL) | Culture supernatant | Beadlyte Human Multi-Cytokine Flex Kits | IL-6, IL-10, IFN-α2, IFN-γ | IL-6 (2.3 pg/mL)  IL-10 (2.3 pg/mL)  IFN-α2 (7 pg/mL)  IFN-γ (2.3 pg/mL) |
| Davies et al., 2011 | Adult | RV-16 (1 MOI) | Culture supernatant | ELISA | IL-6, IL-8, IP-10, TNF-α, IFN-α | IL-6 (4 pg/mL)  IL-8 (7.8 pg/mL)  IP-10 (3.9 pg/mL)  TNF-α (4 pg/mL)  IFN-α (4.9 pg/mL) |
| Iikura et al., 2011 | Adult and Children | RV-14 (1 MOI) | Culture supernatant | ELISA | IL-4, IL-6, IL-10, IL-13, TNF-α, IFN-α, IFN-γ | IL-4 (2 pg/mL)  IL-6 (10.24 pg/mL)  IL-10 (15.6 pg/mL)  IL-13 (0.16 pg/mL)  TNF-α (15.6 pg/mL)  IFN-α (12.5 pg/mL)  IFN-γ (15.6 pg/mL) |
| Sykes et al., 2012 | Adult | RV-16, RV-1B (1 MOI) | Culture supernatant | ELISA | IFN-α, IFN-α2, IFN-β | All 10 pg/mL |
| Pritchard et al., 2014 | Adult | RV-16 (5 MOI) | Culture supernatant | ELISA | IP-10, IFN-α | IP-10 (15.6 pg/mL)  IFN-α (9.7 pg/mL) |
| Upton et al., 2017 | Adult | RV-16, RV-1B (1 MOI) | Culture supernatant | Milliplex map kits | Fractalkine | NS |
| Jurak et al., 2018 | Adult | RV-16 (1 MOI) | Culture supernatant | ELISA | IL-5, IL-13, IFN-γ | NS |
| Hosseini et al., 2021 | Children | RV-1B (20 MOI) | Culture supernatant | ELISA and bead-based multiplex assay | IL-1β, IL-5, IL-6, IFN-γ, IFN-λ | IL-1β (1.8 pg/mL)  IL-5 (1.8 pg/mL]  IL-6 (10 pg/mL)  IFN-γ (1.8 pg/mL)  IFN-λ (10 pg/mL) |

**Supplementary Table 6.** Experimental details of human experimental studies comparing RV-induced cytokine responses in asthmatic vs healthy individuals

| **Study** | **Study Population** | **RV Serotype (Dose)** | **Sample Type** | **Detection Method** | **Measured Cytokines** | **Sensitivity / Detection Limit** |
| --- | --- | --- | --- | --- | --- | --- |
| Fleming et al., 1999 | Adult | RV-16 (2000 TCID_50_) | Nasal lavage, sputum | ELISA | IL-5, IL-6, IL-8, IL-11, RANTES, GM-CSF, IFN-γ | IL-5 (3 pg/mL)  IL-6 (0.7 pg/mL)  IL-8 (10 pg/mL)  IL-11 (8 pg/mL)  RANTES (5 pg/mL)  GM-CSF (0.36 pg/mL)  IFN-γ (3 pg/mL) |
| Kluijver et al., 2003 | Adult | RV-16 (0.6 – 24 x 10^4^ TCID_50_) | Nasal lavage | ELISA | IL-1Ra, IL-1β, IL-8 | IL-1Ra (25 pg/mL)  IL-1β (0.86 pg/mL)  IL-8 (3 pg/mL) |
| DeMore et al., 2009 | Adult | RV-16 (1000 TCID_50_) | Nasal lavage | ELISA & Beadlyte Human Multi-Cytokine Flex Kits | IL-6, IL-8, IL-10, IP-10, MCP-1, RANTES, IFN-α, IFN-γ | IL-6 (2.3 pg/mL)  IL-8 (2.3 pg/mL)  IL-10 (2.3 pg/mL)  IP-10 (6.9 pg/mL)  MCP-1 (6.9 pg/mL)  RANTES (4.1 pg/mL)  IFN-α (7 pg/mL)  IFN-γ (2.3 pg/mL) |
| Laza-Stanca et al., 2011 | Adult | RV-16 | BAL fluid | ELISA | IL-15, IFN-α, IFN-β | IL-15 (0.25 pg/mL)  IFN-α (15 pg/mL)  IFN-β (5 IU/mL) |
| Rohde et al., 2014 | Adult | RV-16 (10000 TCID_50_) | BAL fluid | ELISA & Luminex analysis | IL-8, GRO-α, GRO-β, ENA-78, GCP-2, NAP-2 | IL-8 (3 pg/mL)  GRO-α (5 pg/mL)  GRO-β (10 pg/mL)  ENA-78 (5 pg/mL)  GCP-2 (7.8 pg/mL)  NAP-2 (7.8 pg/mL) |
| Beale et al., 2014 | Adult | RV-16 (100 TCID_50_) | Nasal mucosal fluid | Meso-Scale Discovery array | IL-25 | IL-25 (10 pg/mL) |
| Jackson et al., 2014 | Adult | RV-16 (100 TCID_50_) | Nasal mucosal fluid, bronchial mucosal fluid | Meso-Scale Discovery array | IL-4, IL-5, IL-13, IL-33 | NS |
| Upton et al., 2017 | Adult | RV-16 | BAL fluid | Milliplex map kits | Fractalkine | NS |
| Hansel et al., 2017 | Adult | RV-16 (100 TCID_50_) | Nasal mucosal fluid, bronchial mucosal fluid | Meso-Scale Discovery array | IL-1β, IL-2, IL-4, IL-5, IL-6, IL-8, IL-10, IL-12p40, IL-12p70, IL-13, IL-15, IL-16, IL-17, IL-18, IL-25, IL-33, IFN-β, IFN-λ, IFN-γ, TSLP, TNF-α, GM-CSF, MCP-1, MIP-1α, MIP-1β, RANTES, eotaxin, MCP-4, TARC, MIP-3α, MDC, eotaxin 3, IP-10, ITAC | 1 pg/mL for cytokines, except for eotaxin-3, MCP-1 and IL-33 (3 pg/mL); IL-16 (5 pg/mL); eotaxin, IP-10, TARC, IL-25, TSLP and MIP-1β (10 pg/mL); IFN-β (25 pg/mL); IFN-λ1 (40 pg/mL); and MDC (100 pg/mL) |
| Williams et al., 2021 | Adult | RV-16 (100 TCID_50_) | Nasal mucosal fluid, bronchial mucosal fluid | Meso-Scale Discovery array | MDC, TARC | NS |
